# Role of hormones in bone remodeling in the craniofacial complex: A review

**DOI:** 10.1016/j.jobcr.2023.01.009

**Published:** 2023-01-21

**Authors:** Erin Grinde Tunheim, Hans Erling Skallevold, Dinesh Rokaya

**Affiliations:** a Department of Clinical Dentistry, Faculty of Health Sciences, UIT the Arctic University of Norway, 9037, Tromsö, Norway; b Department of Oral and Maxillofacial Surgery, Faculty of Dentistry, Chulalongkorn University, Bangkok 10330, Thailand; c Department of Clinical Dentistry, Walailak University International College of Dentistry, Walailak University, Bangkok 10400, Thailand

**Keywords:** Hormones, Bone homeostasis, Bone formation, Bone resportion, Bone regeneration, Jawbone, AN, Adiponectin, MAPK, Mitogen-activated protein kinase, RAS, Renin-angiotensin system, ACE, Angiotensin-converting enzyme, ACE2/Ang-(1-7)/MasR, ACE 2/angiotensin-(1-7)/mas receptor, DIZE, Diminazene aceturate, EPO, Erythropoietin, VEGF, Vascular endothelial growth factor, HIF-PHIs, Hypoxia inducible factor-prolyl hydroxylase inhibitors, ES, Estrogen, AD, Androgens, RANKL, Receptor activator of NF-κB ligand, ER, Estrogen receptors, ERα, ER alpha, ERβ, ER beta, GPER1, G-protein coupled estrogen receptor 1, DHT, Dihydrotestosterone, DM, Diabetes mellitus, AGEs, Advanced glycation end-products, RAGEs, Receptor advanced glycation end-products, IGF-1, Insulin-like growth factor-1, PTH, Parathyroid hormone, OT, Oxytocin

## Abstract

**Background:**

Diseases such as periodontitis and osteoporosis are expected to rise tremendously by 2050. Bone formation and remodeling are complex processes that are disturbed in a variety of diseases influenced by various hormones.

**Objective:**

This study aimed to review and present the roles of various hormones that regulate bone remodeling of the craniofacial complex.

**Methods:**

A literature search was conducted on PubMed and Google Scholar for studies related to hormones and jawbone. Search strategies included the combinations (“name of hormone” + “dental term”) of the following terms: “hormones”, “oxytocin”, “estrogen”, “adiponectin”, “parathyroid hormone”, “testosterone”, “insulin”, “angiotensin”, “cortisol”, and “erythropoietin”, combined with a dental term “jaw bone”, “alveolar bone”, “dental implant”, “jaw + bone regeneration, healing or repair”, “dentistry”, “periodontitis”, “dry socket”, “osteoporosis” or “alveolitis”. The papers were screened according to the inclusion criteria from January 1, 2000 to March 31, 2021 in English. Publications included reviews, book chapters, and original research papers; *in vitro* studies, *in vivo* animal, or human studies, including clinical studies, and meta-analyses.

**Results:**

Bone formation and remodeling is a complex continuous process involving many hormones. Bone volume reduction following tooth extractions and bone diseases, such as periodontitis and osteoporosis, cause serious problems and require a great understanding of the process.

**Conclusion:**

Hormones are with us all the time, shape our development and regulate homeostasis. Newly discovered effects of hormones influencing bone healing open the possibilities of using hormones as therapeutics to combat bone-related diseases.

## Graphical abstract

**Figure:**
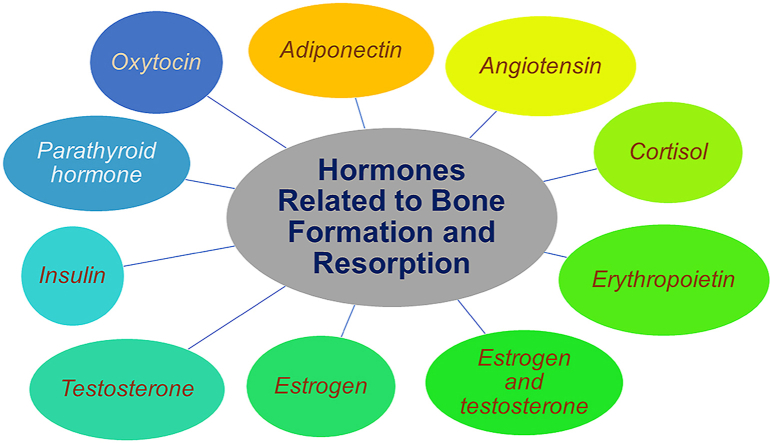

## 1 Introduction

Craniofacial growth is a highly complex process. Aside from the calvarium and facial bones, the maxilla is characterized by independent vertical growth and contains dentition, despite being attached to the cranium. Most interestingly, the mandible exclusively articulates at the temporomandibular fossae and is characterized by hanging in a sling of muscles and containing the dentition. The latter property makes both the maxilla and the mandible the bones with the highest rate of remodeling to accommodate tooth eruption and mastication. Several hormones are implicated to play a role in bone physiology, with changes in these hormones’ levels, changes in the bone can also be observed.^1^^,^^2^

**Fig. 1:**
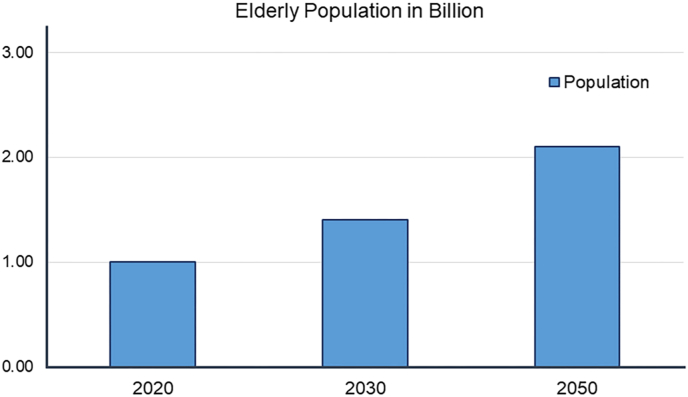
Globally, the number of older adults may be expected to reach 1.5 billion by 2050, and the prevalence of periodontitis and osteoporosis as well. That is a fivefold increase from 1990.^3^ Ways to manage and prevent these diseases from developing are warranted.

Osteoporosis and periodontitis are global diseases affecting bone highly correlated with increasing age and global burden as shown in Fig. 1.^3^ Increased risk of cardiovascular disease^4^ aspiration pneumonia^5^ and severe outcome of COVID-19^6^ are associated with periodontitis. By 2040, the prevalence of periodontitis is expected to have increased by 50% among older adults 65 years and older.^7^ Severe bone loss and loss of teeth occur if left untreated, making implant placement challenging. The number of dental implants placed is rapidly growing,^8^ and their success rate depends on the available bone to be inserted into.^9^ Osteoporosis' impact on the survival of dental implants is debated, however, observations point toward these patients possibly having a slight disadvantage for their implants’ survival.^10^^,^^11^ The gold-standard technique to rebuild adequate bone levels is the autologous bone graft.^12^ However, harvesting the graft is invasive and limited by available donor bone, increased morbidity, and post-operative pain.^13^^,^^14^ Researchers are continually developing new methods to prevent bone loss and to rebuild bone in the jaw. Hormones may be a direction to explore for such new methods as several hormones are implicated in osteoporosis, periodontitis, and bone remodeling.

While hormones are used for a wide array of conditions in medicine, little is known about hormones in dentistry and their possible therapeutic applications. Hence, it is important to review the various hormones that regulate bone formation and resorption implications on the jawbone and the diseases. This study aimed to review and present the roles of various hormones that regulate bone remodeling of the craniofacial complex.

## 2 Method

A literature search was conducted on PubMed and Google Scholar for studies related to hormones and jawbone as shown in Table 1. Search results were screened for relevance; publications describing or investigating hormone(s) effects on jawbone in relation to dentistry. The publications’ cited papers were screened for relevance, as well, and included if fitting inclusion criteria. Additional inclusion criteria included full-text availability (Table 1).

**Table 1:** Literature search strategy and study selection criteria.

	Literature Search Strategy and selection criteria
A	A literature search was conducted on PubMed and Google Scholar for studies related to hormones and jawbone. Search strategies included the combinations (“name of hormone” + “dental term”) of the terms: “hormones”, “oxytocin”, “estrogen”, “adiponectin”, “parathyroid hormone”, “testosterone”, “insulin”, “angiotensin”, “cortisol”, and “erythropoietin”, combined with a dental term “jaw bone”, “alveolar bone”, “dental implant”, “jaw + bone regeneration, healing or repair”, “dentistry”, “periodontitis”, “dry socket”, “osteoporosis” or “alveolitis”. The papers were screened according to the inclusion criteria from January 1, 2000 to March 31, 2021 in English. Publications included reviews, book chapters and original research papers; *in vitro* studies, *in vivo* animal, or human studies, including clinical studies, and meta-analyses.
B	English language, publication date from January 1, 2000 to March 31, 2021. 1. Publications included reviews, book chapters and original research papers; *in vitro* studies, *in vivo* animal, or human studies including clinical studies, and meta-analyses. 2. Where no studies could be found on jawbone specifically, we included studies to elucidate the basic mechanisms and relevant medical studies, by removing “jaw” from the search words. 3. Case studies, letters, viewpoints, editorials, publications providing irrelevant or unusable data, lack of full text availability, and non-English language articles were excluded.

### 2.1 Hormones related to bone remodeling

**Fig. 2:**
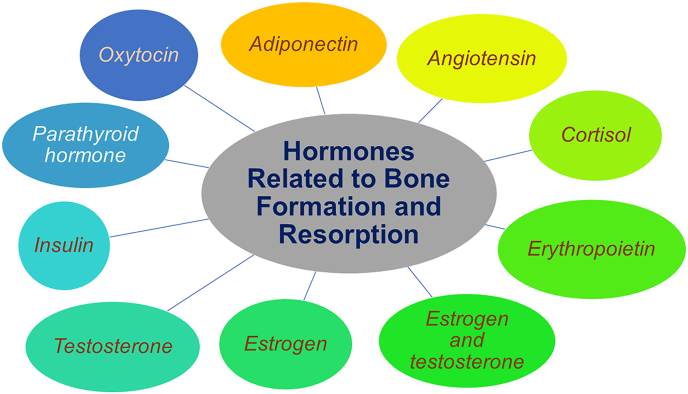
Hormones related to bone formation and resorption.

Hormones are substances produced in the endocrine glands of the body and released to the bloodstream and further carried to specific target cells, where their effects are exercised. Such endocrine glands include the pituitary gland, adrenals glands, parathyroid glands, and more.^15^ Hormones may act as messengers or something as complex as coordinators of various essential processes such as blood volume and blood pressure regulation, development, and reproduction.^16^^,^^17^ Following nine hormones were found to be involved in the bone remodeling of the jaw bone (Fig. 2).^18^, ^19^, ^20^, ^21^, ^22^, ^23^, ^24^, ^25^, ^26^, ^27^

### 2.2 Adiponectin

Adiponectin (AN), a hormone produced by adipose tissue, is typically implicated in the regulation of blood glucose and the oxidation of fatty acids. However, the hormone has many additional biological functions.^28^ Recently, AN has been found to play a potential role as a positive bone mass regulator,^29^ an angiogenesis stimulator, and an osteoclast suppressor.^30^ AN interacts with the cementoblasts (OCCM-30) affecting the cell migration, proliferation and cementogenesis partly through the Mitogen-activated protein kinase (MAPK) signaling pathway.^31^ Activation of AN-mediated migration and proliferation was results after the inhibition of P38, ERK1/2 and JNK in various degrees, whereas mineralization was increased by MAPK inhibition in varying degrees. These all favorably increases the cell proliferation and cementogenesis. Hence, AN regulates bone formation and serves as a potential application in orthodontics**.**

Administration of AN in rabbits has resulted in increased mineral content, increased mechanical strength, and a coarser bone morphology locally, suggesting new and accelerated bone formation.^32^ The rabbits in the study were undergoing a procedure known as distraction osteogenesis, a surgical method of elongating bone by cutting it and slowly pulling the pieces apart using a mechanical device over time. It was evaluated that the key steps of AN-induced bone regeneration in the surgically made gaps involved the recruitment and clonal expansion of bone-forming cells under mechanical stimulation.^18^^,^^33^^,^^34^ In addition, two AN-receptors have been identified to be expressed by osteoblasts, suggesting AN's direct functions in bone metabolism,^35^ by promoting proliferation and stimulating bone formation.^32^ Bone tissue is dependent on adequate blood circulation; therefore, angiogenesis is an important component of bone regeneration and function, as well. The AN has proven to influence cellular responses in endothelial cells in an ischemic state to promote angiogenesis.^36^ Indeed, Ouchi and collaborators reported increased angiogenesis in mouse and rabbit models following the administration of AN.^30^ Repeated local administration of AN in rats showed the potential of AN as a potential therapeutic to prevent orthodontic treatment relapse, as reported by Haugen et al.^37^

AN has been found to heal the periodontitis partly due to its action in inflammation and bone.^38^ APN can upregulate the enamel matrix derivative-induced expression of growth- and osteogenesis-associated factors, and it has the potential for bone regeneration.^39^ Hecne, APN and its agonists can be used in the treatment of periodontitis. Since it is not feasible to raise the circulating APN level in humans (normal physiological level of APN is 3–30 μg/ml), complicated post-transcriptional modification can be done.^38^

### 2.3 Angiotensin

The human body controls blood pressure and fluid volume by an endogenous hormone system known as the renin-angiotensin system (RAS). Hypertension is a pathological consequence that may occur due to RAS dysfunction. Angiotensin is formed in the liver and released in an inactive state, which is then split by the enzyme renin and converted to angiotensin I, and further split into angiotensin II by the angiotensin-converting enzyme (ACE).^40^ Angiotensin is important in volume and blood pressure control.^41^

Angiotensin can be generated by endothelial cells outside of the RAS. Researchers reported that in co-cultures of osteoclasts with osteoblast cells, angiotensin I activated bone resorption. This finding may show that RAS could play a role in bone resorption control.^42^ Saravi et al.^43^ concluded that inhibition of RAS in an animal model could lead to a reduction in periodontal bone loss and reduction in inflammation intensity. Whether or not various tissues and organs of rats can generate angiotensin independently of circulating RAS has been reported.^19^ Interestingly, gingival tissue of rats express RAS locally, making it possible to produce angiotensin II *in vitro*.^19^ Angiotensin may influence contribute to bone degradation in periodontitis but increased renin production may increase the risk of periodontitis. Santos et al.^19^ discussed the mechanism behind the increased risk to involve bacteria stimulating the expression of the gingival RAS, resulting in a proinflammatory environment by increased angiotensin II-levels which could contribute to the bone loss experienced in periodontitis. Understanding the inflammatory effect of RAS can provide a unique perspective for clinical research and treatment of periodontitis.

RAS has been used to treat inflammatory disorders.^44^ The ACE 2/angiotensin-(1–7)/Mas Receptor (ACE2/Ang-(1–7)/MasR) axis is found to be associated with bone remodeling in osteoporosis. Treatment of bone cells with Ang-(1–7) or diminazene aceturate (DIZE) stimulated osteoblast ALP, matrix synthesis, upregulated osterix, osteocalcin and collagen type 1 transcription, reduced IL-6 expression, and decreased osteoclast differentiation, RANK and IL-1β mRNA transcripts, and IL-6 and IL-1β levels, in a MasR-dependent manner.^45^ In vivo, Ang-(1–7) and DIZE decreased alveolar bone loss through improvement of osteoblast/osteoclast ratio. A-779 reversed such phenotype. ACE2/Ang-(1–7)/MasR axis activation reduced IL-6 expression, but not IL-1β. ACE2 and MasR were also detected in human gingival samples, with higher expression in the healthy than in the inflamed tissues. These findings show that the ACE2/Ang-(1–7)/MasR is an active player in alveolar bone remodeling.

### 2.4 Cortisol

Cortisol, a steroid hormone produced by the adrenal glands, is released into the bloodstream, passes through the cell membrane, and translocated to bind to the cell nucleus receptor proteins, triggering changes in gene expression. Stress triggers the release of cortisol. Typical examples of stress triggers include fear, anxiety, hemorrhage, pain, low blood glucose, illness, and starvation. To endure stress, muscle, liver, and adipose tissue empty their storage of nutrients as a response to cortisol. Chronically elevated cortisol levels cause muscle and bone damage, impaired endocrine and immune function.^16^ Stress is a factor leading to the onset of illness.^46^

Tissue repair is reportedly impaired by chronic and acute stress in rats.^47^ Previous studies have reported that chronic stress worsens healing.^48^ and the formation of the bone matrix and collagen fibers, and a decreased number of osteoblasts.^20^ Experimental models involving the periodontium of rats have shown that stress increases susceptibility and worsens periodontitis, PD.^49^ Studies have indeed shown the potential of the association of stress markers, inflammation, and PD.^50^^,^^51^ Similarly, the progression of periodontitis is related to the stress as a factor in mind.^52^ Is it indicated that stress appears to be associated with microbial colonization between highly stressed patients and non-stressed patients. However, the salivary cortisol concentration is not associated with stress.^53^ Therefore, stress and subsequent cortisol increase may contribute to the progression of periodontitis.

As cortisol disturbs healing, it has also been found that chronic stress has been proven to disturb the initial repair process in the rat mandible following implantation. If this is true also in human patients, if patients’ chronic stress levels affect the initial phases of osseointegration, it is left for future research.^54^

Cortisol suppress the resorptive action of parathyroid hormone, which stimulates the proliferation of progenitor cells and favors their differentiation towards osteoclasts.^55^ Hence, cortisol inhibits bone resorption *in vitro* by limiting the ability of precursor cells to form osteoclasts.

### 2.5 Erythropoietin

Erythropoietin (EPO), N-linked glycoprotein consisting of 166 aa, well-known hormone released from the kidneys for its red blood cell-producing effects.^56^ EPO production is activated by hypoxia and is regulated via an oxygen-sensitive feedback loop.^57^ EPO can increase bone formation indirectly by increasing vascular endothelial growth factor (VEGF) expression.^16^^,^^58^ Through osteoblast-osteoclast communication pathways, EPO often indirectly activates osteoblast differentiation. These experiential findings are important in the understanding of mediated bone remodeling and may contribute to the treatment of bone defect growth.^59^ EPO can contribute to bone formation both directly by communication pathways and indirectly by VEGF.^58^^,^^60^

EPO can be used to combat periodontitis. Holstein et al.^21^ experimented by delivering EPO to dental extraction sockets and observed that EPO significantly promoted new bone formation. Suggested by the mechanisms of inhibiting pro-inflammatory pathways and apoptosis, improved vascularization, and enhanced osteoblast formation. Aslroosta et al.^61^ evaluated the effect of EPO on the improvement after phase I periodontal treatment, and they found that there was a significant reduction in calculus and periodontitis in the test group. They suggest that EPO gel can improve clinical inflammation and calculus in periodontitis. Hence, EPO can be used as an adjunct to non-surgical periodontal therapy as it provides a promising results in moderate to severe chronic periodontitis.

EPO can be used in regenerative medicine for the treatment of tissue de-regeneration disorders.^62^^,^^63^ EPO acts both as a peptide hormone and hematopoietic growth factor, stimulating bone marrow erythropoiesis.^57^ EPO acts via its homodimeric erythropoietin receptor that increases cell survival and drives the terminal erythroid maturation of progenitors BFU-Es and CFU-Es to billions of mature RBCs. This pathway activates the multiple erythroid transcription factors, such as GATA1, FOG1, TAL-1, EKLF and BCL11A, and leads to the overexpression of genes encoding enzymes involved in heme biosynthesis and the production of hemoglobin.^57^ Hence, these properties of EPO can be used to treat anemias associated with chronic kidney diseases and blood disorders such as anemia.^62^^,^^63^ EPO affect osteoblast and osteoclast activity during EPO-stimulated erythropoietic response stimulate the debate on the relative efficacy and safety of EPO vs hypoxia inducible factor-prolyl hydroxylase inhibitors (HIF-PHIs) for patients who require long-term treatment for anemia.^62^

### 2.6 Sex hormones

In both sexes, steroid hormones regulate skeletal preservation and maturation, and the impact of gonad insufficiency on skeletal integrity has been widely recognized.^64^ Ovariectomy and orchiectomy have been shown to cause condylar bone loss in mandibular condylar bone.^65^ Estrogen (ES) is a group of female sex hormones responsible for the development of female sexual characteristics and the reproductive system. ES comprises a group of steroid hormones, typically estradiol, estrone, and estriol,^66^ and AD; testosterone, dihydrotestosterone, and androstenedione. ES is produced in a lesser amount in males, where androgens (AD), the male sex hormones, dominate. Steroid hormones are synthesized in several endocrine tissues, such as the ovaries in females and in the testicles of males. They can bind to the receptors in the plasma membrane of cells and cause rapid effects.

Estrogen plays an important role in the growth of bone, maturation of bone and regulation of bone turnover in adult bone.^67^^,^^68^ During bone growth estrogen is needed for proper closure of epiphyseal growth plates both in females and in males.

#### 2.6.1 Estrogen

ES regulates bone remodeling by modulating the expression of receptor activator of NF-κB ligand (RANKL), an essential cytokine for bone resorption by osteoclasts.^69^ The increase in bone resorption observed in states of estrogen deficiency in mice is mainly caused by lack of ERα-mediated suppression of RANKL expression in bone lining cells. ES exerts its action by binding to its multiple receptors in the cell membrane and cytoplasm. Until now at least three estrogen receptors (ER) have been reported: ER alpha (ERα), ER beta (ERβ), and G-protein coupled estrogen receptor 1 (GPER1) also known as GP30.^67^ ES deficiency leads to increased osteoclast formation and enhanced bone resorption also in young skeleton estrogen.

In menopause, ES deficiency induces both cancellous and cortical bone loss.^68^ There can be increased bone resorption in cancellous bone leads to general bone loss and destruction of architecture because of penetrative resorption and microfractures.^68^ In cortical bone, at first estrogen causes endocortical resorption and then increases intracortical porosity. These lead to decreased bone mass, disturbed architecture and reduced bone strength. At cellular level, ES inhibits differentiation of osteoclasts thus decreasing their number and reducing the amount of active remodeling units. This is mediated through cytokines, IL-1 and IL-6. It is still not clear if the effects of ES on osteoblasts is direct or is due to coupling phenomenon between bone formation to resorption.

Further more, ES can cause changes at the level of gene expression, through nuclear receptors in the cell nucleus, though these effects are slow.^16^ The ability of estrogen to signal not only through its nuclear receptors, ERα and ERβ, but also through membrane-bound receptors, e.g., GPER1/GP30, the possible crosstalk with other signaling pathways, e.g., BMP4 signaling pathway, epigenetic regulation of estrogen receptor itself, and recruitment of coactivators that are able to modify DNA methylation and histone arrangement might shed a light on explaining the wide effects of estrogen in bone.^67^ In addition, Sundeep et al.^70^ reported that estrogen inhibits bone resorption by direct action on osteoblasts, through estrogen modulation of osteoblasts and osteocyte and T-cells regulation of osteoclast differentiation and activity.

Ayed et al.^71^ investigated the association of osteoporosis and the progression of PD in postmenopausal females and concluded that OP is a risk factor for PD. Indeed, among women with early menopause, increased bleeding and bone loss have been observed, as well as increased levels of CRP, indicating systemic inflammation. These findings suggest increased severity or risk of periodontitis among postmenopausal women.^72^ Previously, there has been debate among researchers on whether oral contraceptives may increase the risk of or worsen PD. According to Preshaw,^22^ it suggests that oral contraceptives on the market today no longer place users at any risk of PD.

#### 2.6.2 Testosterone

Testosterone, the most well-known androgen, is mainly anabolic in effect.^73^ Testosterone can be reduced by a cytoplasmic enzyme called 5-alpha-reductase, to dihydrotestosterone (DHT). DHT binds to the same androgen receptor 2.5 times stronger than testosterone and thus shows increased androgenic potency.^74^ Testosterone is responsible for the development of male reproductive organs and sexual characteristics, including increased bone and muscle mass.^75^

Testosterone treatment has been found to improve the areal and volumetric bone mineral density at the spine and hip.^76^, ^77^, ^78^, ^79^, ^80^ Testosterone regulates the bone remodeling either directly or via aromatization to estradiol by activating sex steroid receptors in bone cells.^81^ Furthermore, testosterone increases muscle mass which may have indirect anabolic effects on bone mass.^82^ Recently, testosterone replacement therapy is an also useful tool for managing clinical symptoms caused by hypogonadism. Men with osteopenia and osteoporosis.^80^

The administration of testosterone has shown to affect the human gingiva by increasing the growth rate of oral bacteria, which is causally considered to be related to periodontal inflammation. It has been reported that human gingival tissues metabolize testosterone^83^ with conversion to DHT.^84^ Interestingly, inflamed tissue, including inflamed gingival tissue, has a two to three-fold increase in DHT receptors.^85^ Testosterone deficiency in older men is one of the risk factors for OP. Androgens can be converted to ES through a process known as aromatization.^86^ It has been shown in previous *in vitro* studies that androgen can promote pre-osteoblast proliferation and converted ES can suppress osteoclast development. Human studies among elderly men, both androgen and ES, are necessary for the formation of bone. ES is required for the suppression of bone resorption. While androgens are essential for older men in the prevention of OP and its complications.^23^ According to Kellesarian et al.,^87^ four studies showed no correlation between serum testosterone and chronic periodontitis, and two studies with a positive correlation. However, their limits to the evidence and further longitudinal studies are needed.

### 2.7 Insulin

Insulin is a peptide hormone developed and secreted by beta cells in the Langerhans islets of the pancreas. The hormone is essential in maintaining normal blood glucose levels by facilitating the absorption of glucose into cells. Insulin is, therefore, a vital component of anabolism.^88^

Diabetes mellitus (DM), a disease of reduced production or sensitivity to insulin, reports delayed bone formation and impaired fracture healing. DM increases and prolongs inflammation-promoting osteoclast differentiation. Subsequently, the balance of bone remodeling shifts towards a resorbing tendency. Indeed, it has been reported that insulin activates osteoblast differentiation, reduces apoptosis of osteoblasts, and reduces osteoclast activity.^24^ Kido et al.^89^ performed an experiment on diabetic rats and reported that an association between periodontitis and diabetes is evident. They also reported that diabetes can induce abnormal proliferation of gingival fibroblasts. Insulin resistance plays a role in the progression of periodontitis in diabetic patients. They concluded that delayed gingival wound healing in diabetic rats was caused by impaired proliferation and migration of fibroblasts. Dysfunction of fibroblasts may be caused by high glucose-induced insulin resistance via oxidative stress.^89^ It is evidence of DM type II and stimulation of impaired cellular function and hemostasis that lead to periodontitis. Kinane and the group found that advanced glycation end-products (AGEs) are produced by the interaction between sugar, aldoses, and lipid. The DM type II patients do have both hyperglycemia and hyperlipidemia which is perfect for producing AGEs. On the surface of macrophages, there is a receptor called receptor advanced glycation end-products (RAGEs) which will interact with AGEs and promotes transcription factor (NK-FB). This interaction induces transcription and causes macrophages to produce proinflammatory cytokines, for example, interleukin 1, interleukin 6, and tumor necrosis factor-alpha, and leads to periodontal ligament destruction. There are also been found that macrophages will delay wound healing.^90^

A growth factor so similar to insulin in the structure that it has been named insulin-like growth factor-1 (IGF-1) can be secreted from the liver by the stimuli of human growth hormone, but also by bone cells.^91^ IGF-1 is critically involved in bone growth during puberty and throughout life.^92^ IGF-1 has also proved to, in combination with other growth factors, accelerate bone formation around dental implants in rabbits.^93^^,^^94^ Perhaps due to their structural similarity, insulin itself has bone-promoting properties with osteoblasts expressing insulin-receptors – possibly promoting osteoblast differentiation.^91^ The synthesis of IGF-1 is controlled by GH in chondrocytes, whereas it is regulated by parathyroid hormone (PTH) in osteoblasts. IGFs are expressed by human osteoblasts. The IGF-1 induce differentiated osteoblastic activity without influencing stromal cell differentiation directly into mature osteoblast. Therefore a low decline in the expression of IGF-1 is considered essential for the occurrence of cellular apoptosis and to facilitate the differentiation of osteoblast.^95^

An animal study^96^ explored the role of insulin in the integration of titanium implants in rat tibias in which 3 groups of rats were involved: diabetes-induced rats, insulin-injected rats, and healthy controls. Up to three weeks after implant insertion, it was found that delayed healing was evident in the diabetes rats, which showed improvement after insulin injection. While the controls and insulin-treated rats had similar amounts of bone healing. It would be of interest to conduct a similar study on the jawbone. Insulin is involved in bone healing, although the mechanisms are not entirely elucidated. Understanding the mechanisms more thoroughly may contribute to a better understanding and possibly a future local insulin treatment of periodontitis worsened by DM.

### 2.8 Parathyroid hormone

Parathyroid hormone (PTH) regulates the calcium homeostasis of the body.^97^ PTH is secreted from four glands located behind the thyroid gland, where they monitor and regulated serum levels of calcium. When levels are low, PTH is released into the bloodstream to release calcium from the body's largest reservoir, the skeleton, by bone resorption.^98^

Recent clinical and pre-clinical studies indicate that PTH increases the bony density of the jaw and enhances soft tissue healing and bone filling after tooth extraction.^99^ In rat models with PD, PTH was reported to have an anti-inflammatory effect.^100^ Inflammation in the gingiva was significantly reduced, and bone loss was suppressed by PTH.

Animal and human studies have shown that PTH administration leads to increased bone formation through an increase in osteoblast number and surface, as well as an increase in mineralized matrix deposition through effects on proliferation of precursors, suppression of apoptosis, and activation of lining cells.^101^^,^^102^ In addition, *in vitro* and *in vivo* studies showed that PTH directly activates survival signaling in osteoblasts; and that delay of osteoblast apoptosis is a major contributor to the increased osteoblast number, at least in mice.^102^

Ji-Hye Kim intermittently administered PTH DM-rats with periodontitis and found that such an administration regimen of PTH reduced alveolar bone loss and increased bone formation.^103^ This finding suggests that PTH administration counteracted bone loss, as promoted by DM, by inducing bone formation. A combination of the protein SDF-1alpha and PHT showed enhanced bone formation, SDF-1alpha also plays a promoting role in the regeneration of PDL.^103^ Several studies have examined the impact of PTH on dental implant stability and integration in bone. Bellido et al.^104^ induced artificial osteoporosis in rabbits and measured general bone loss and reduced mineral content in their jaws. However, administration of PTH almost completely reversed these negative findings, and restored the jawbone to almost normal levels. A study on mongrels found increased levels of bone remodeling around dental implants inserted in the mandible, in the group administered PTH.^25^ An application perhaps close to clinical utilization is presented in a paper investigating PTH-coated titanium dental implants in rats.^105^ The results were promising, with increased bone formation around the PTH-coated implants. Therefore, these results together suggest that PTH might represent a future therapy for improving the integration of dental implants in humans. However, the frequency of PTH administration varies among studies, therefore a priority should be to find the optimal frequency and dosage to improve bone growth.^97^

### 2.9 Oxytocin

Oxytocin (OT) is produced in the hypothalamus and excreted via the pituitary gland.^106^ The hormone acts on the mammary gland and uterine muscles. The hormone can induce uterine contractions during pregnancy, contributing to childbirth. During lactation, OT causes milk release.^16^ Bone cells express OT receptors and OT is involved in the process of bone remodeling,^107^ as it has been reported to reduce the resorption of bone and cause a relative increase in the formation of bone.^108^ In addition, OT promotes osteoblasts differentiation and function which leads to an increased bone formation and an improvement of bone microarchitecture.^109^ Low oxytocin serum levels is associated with postmenopausal osteoporosis.^110^^,^^111^ and have been found to play a role in skeletal homeostasis.^108^ Furthermore, intramuscular injection of OT has been shown to promote bone growth in rats, with consequent alterations in serum levels of calcium, RANKL, and OPG.^112^ OT-treated rats have shown increased levels of osteocalcin and a significant increase in alveolar bone formation.^113^ Osteocalcin is synthesized by osteoblasts and can be used as an indicator of bone remodeling and mineralization of the bone matrix.^114^^,^^115^

Treatment with OT is shown to result in an increase in levels of intracellular calcium and to regulate stimulation of osteoblast formation and thus bone formation in rats. Additionally, deletion of the OT-receptor in mice resulted in the development of OP.^108^ Systemic OT has been investigated regarding the OP with positive results, such as improved peri-implant bone healing in the distal femoral metaphysis.^116^ In OP, the favored osteoclast activity has been suggested to be an implication of a lack of OT.^115^ A study done by Jee et al.,^26^ showed that OT stimulates a reduction of bone resorption and yields a positive bone balance during the process of alveolar bone healing in female rats.

## 3 Discussion

There is plenty of available research about hormones in the medical literature, however, the literature is scarce in the field of dentistry. This review aimed to shed light on available research on the topic of hormones and dentistry regarding their effects on the jawbone and related oral disease. This is the first article that reviewed various hormones that regulate bone formation and resorption implication on the jawbone and the diseases, and presented the role of hormones on bone remodeling and metabolism. Additionally, the authors have selected three diseases to discuss how they are influenced by hormones: osteoporosis, periodontitis, and dry socket. Table 2 shows the summary of the hormones’ impact bone healing, by a variety of mechanisms.

**Table 2:** Summary of the hormones that beneficially impact bone healing, by a variety of mechanisms.

Hormones	Effects on bones
Oxytocin, estrogen, testosterone, adiponectin, parathyroid hormone, insulin, and erythropoietin.	Increase influences the bone balance positively.
Cortisol and angiotensin	Increase results in bone loss.

In addition, abnormalities in both local and endocrine regulation of growth plate physiology cause various problems in human skeletal growth. Hence, knowledge of these pathways has therapeutic potential for sustaining or even augmenting linear growth.^117^

In general, all of them may have therapeutic potential. Not all of the hormones had available research on jaw bone specifically. However, the basic hormonal mechanisms may be transferable to involve jaw bone. Some major differences need to be considered, such as a high degree of remodeling rate and growth^118^ of the jaw bone.

Development of therapeutic hormone-based treatment to combat bone loss in the jaw are supported by promising animal studies for both PTH-coated implants^105^^,^^119^ and adiponectin for orthodontic treatment.^37^ The authors’ next step could be human clinical trials. PTH is interesting as it is safely used in the treatment of osteoporosis already and therefore has an established safety profile for that indication.^120^^,^^121^ Adiponectin is promising as it may be able to prevent post-treatment movement of orthodontically treated teeth, although human clinical trials with long follow-up times are necessary to evaluate this potential usage.

As with any treatment, hormone treatment is not free of risk. Future research needs to focus on finding the optimal therapeutic dosage for bone healing with as few side effects as possible. For example, the use of estrogen carries risks of, breast cancer,^122^ and thromboembolism.^123^ As estrogen illustrates, hormones are agents with systemic effects, able to affect several different tissues in a variety of ways. Local treatment with hormones may therefore be more advantageous than systemic administration. The ideal scenario would be the possibility of “naturally” enhancing the hormone levels to achieve a clinical outcome. For example, oxytocin levels rise by prolonged hugging or intimate contact.^124^ The hormone levels needed to achieve desired clinical outcomes may be much higher than physiologically produced amounts. Another disadvantage to keep in mind is that hormones are relatively slow-acting agents, meaning that the patient may not experience immediate clinical improvement. Additionally, the patient may require frequent administrations to achieve desired clinical effects. Thus, with long treatment times and frequent administrations, hormone treatment may not be cost efficient, although there are still insufficient data to consider this aspect as of now. Some possible benefits include convenient monitoring of hormone levels in the blood.^87^ Thus, the dosage can be individually regulated for optimal effect. Hormone treatment may be especially suited for individuals with conditions resulting in or from hormonal imbalance, such as osteoporosis. Therefore, finding the optimal dosage and frequency of hormone administration for bone repair is would be beneficial to achieve a therapeutic window in a clinical setting.

The role of estrogen in osteoporosis is well-known, however, more surprising was the influence of hormones on periodontitis and dry socket. A suggested correlation of estrogen and dry socket^125^ has been reported with a plausible mechanism of estrogen degrading the healing blood clot in the postoperative alveolus. This is debated as somewhat conflicting reports exist.^22^^,^^126^ Oral contraceptives and possibly menstruation where higher levels of female sex hormones, predominantly estrogen may increase the risk for dry socket. The risk of AO may not be apparent with today's contraceptives.^22^ However, with the increasing number, or rather the awareness of them, of gender, transitioned men-to-females, a group we know little about may indeed require attention. This group of transgenders requires regular injections of estrogen, at much higher levels than that found in commercial contraceptives.^127^ One may speculate that a much higher risk of postoperative complications such as dry socket exists if estrogen is injected around the same time as dental extraction. Little research exists on this group of patients in dentistry and warrants further research. Dentists may consider asking this group of patients about their injection schedule as to not risk possible post-operative complications.

Some evidence suggests a link between low testosterone levels and risk of osteoporosis.^23^ Some authors also suggests a correlation between testosterone and periodontitis, however, conflicting results exist in the literature.^87^ An increased risk of bone fractures in men with testosterone deficiency, usually older adults, is apparent,^128^ however, no studies report fracture incidence on specific bone sites, such as the mandible. A study^128^ showed that testosterone supplementation can prevent this could not be concluded however, sexual function and quality of life could improve. The authors did explore another aspect of male sexual function and dentistry. Low-grade inflammation by periodontitis has been reported to be associated with erectile dysfunction. Interestingly, patients with erectile dysfunction also experienced improvement after periodontal treatment.^129^ The mechanism is unknown; however, the field of periodontitis and the male sex seems to contain more mysteries than expected.

The field of hormones and dentistry remains scarcely researched. Most assumptions and hypotheses are extrapolated from the medical literature where hormone research is more abundant. Some diseases of the oral cavity commonly encountered are possibly influenced by hormones. Certain populations requiring hormone replacement therapy, such as transgenders, and the dental consequence of such therapy is largely unknown. Transitioned men-to-females, require regular injections of estrogen, at much higher levels than found in commercial contraceptives.^127^ Little research exists on this group of patients in dentistry and warrants further research. . Hormone treatment in dentistry is far from common practice, however, some hormones are well researched, and some are regularly used in other medical settings. Hormone treatment in dentistry may be beneficial for some oral diseases, in relation to orthodontic retention, improved bone healing and dental implant osseointegration. More research is needed before therapeutic usage in dentistry can be implemented safely and effectively.

## 4 Conclusion

Bone formation and remodeling is a complex continuous process involving many hormones. Bone volume reduction following tooth extractions and bone diseases such as periodontitis and osteoporosis cause serious problems and require a great understanding of the process. Hormones shape our development and regulate our homeostasis. Newly discovered effects of hormones influencing bone healing open the possibilities of using hormones as therapeutics to combat bone-related diseases. As the hormones may have a multitude of different effects, the safety and regimens of administration regarding dosing, location, and frequency need to be assessed and more studies and clinical trials are needed.

## Data availability

The data used to support the findings of this study are available from the corresponding author upon reasonable request.

## Funding

This research did not receive any special grant from funding agencies in the public, commercial, or not-for-profit sectors.

## Declaration of competing interest

The authors declare no conflicts of interest.
